# Fertility‐Related Distress in Young Adult Survivors of Childhood Cancer: Prevalence and Key Predictors

**DOI:** 10.1002/cam4.72470

**Published:** 2026-11-14

**Authors:** Anu Haavisto, Kirsi Jahnukainen, Poorna Anandavadivelan, Emma Hovén, Lena Wettergren, Claudia Lampic

**Affiliations:** ^1^ Childhood Cancer Research Unit, Department of Women's and Children's Health Karolinska Institutet Stockholm Sweden; ^2^ Department of Psychology, Faculty of Medicine University of Helsinki Helsinki Finland; ^3^ The Faculty of Human and Social Sciences Åbo Akademi University Vasa Finland; ^4^ Division of Haematology‐Oncology and Stem Cell Transplantation, Children's Hospital University of Helsinki and Helsinki University Hospital Helsinki Finland; ^5^ Department of Neurobiology, Care Sciences and Society, Division of Nursing Karolinska Institutet Stockholm Sweden; ^6^ Department of Women's and Children's Health Uppsala University Uppsala Sweden; ^7^ Uppsala University's Centre for Women's Mental Health – WOMHER Akademiska Sjukhuset Uppsala Sweden; ^8^ Department of Public Health and Caring Sciences Uppsala University Uppsala Sweden; ^9^ Department of Women's and Children's Health Karolinska Institutet Stockholm Sweden; ^10^ Department of Psychology Umeå University Umeå Sweden

**Keywords:** cancer survivorship, childhood cancer, fertility distress, psychological distress, reproductive health, self‐report, young adults

## Abstract

**Introduction:**

Impaired fertility can occur as a late effect of childhood cancer treatment. This study aimed to assess the prevalence and predictors of fertility‐related distress in young adult childhood cancer survivors, compared to a general population sample.

**Methods:**

This nationwide, population‐based cross‐sectional study included 1333 women (67% response rate) and 1213 men (54%) aged 19–40, who had been treated for cancer in Sweden between ages 0 and 17. Eligible survivors were identified through the National Quality Registry for Childhood Cancer in Sweden. A comparison group was randomly selected from the Swedish Population Registry, comprising 493 (51% response rate) women and 326 (34%) men. The study combined clinical registry data and self‐report measures. The main outcome was high fertility‐related distress assessed with the six‐dimension Reproductive Concerns After Cancer (RCAC) scale, with high distress defined as a mean score above 4. The comparison group completed only the three RCAC dimensions relevant to adults without a history of cancer. Group comparisons were undertaken using logistic regression, with analyses stratified by sex.

**Results:**

A total of 44% of female and 23% of male survivors reported high fertility‐related distress in at least one RCAC dimension. Distress was most prevalent in the Fertility potential dimension, and both female and male survivors were significantly more likely to report high distress regarding fertility potential than women and men in the comparison group. Among survivors, high fertility‐related distress across several RCAC dimensions was associated with not having children, the desire for (additional) children, having undergone intensive cancer treatments or fertility‐preservation procedures, receiving ongoing hormone replacement therapy, and experiencing concurrent anxiety and depressive symptoms.

**Conclusion:**

The results underscore the need to address fertility‐related concerns in the follow‐up care of childhood cancer survivors.

## 1 Introduction

Advances in cancer treatment have led to a growing number of children surviving cancer. In Europe alone, there are an estimated 500,000 childhood cancer survivors (CCS) [1]. In the long term, CCS may face serious late effects from their treatment, of which one major concern is endocrine complications [2, 3]. In males, this can manifest as testosterone deficiency and impaired spermatogenesis [4, 5], and in females as premature ovarian insufficiency [6, 7], both of which may lead to impaired fertility in adulthood [8]. The risk of endocrine complications is higher following more intensive cancer treatment regimens, such as irradiation to the gonads, high cumulative doses of alkylating chemotherapy, and conditioning therapy for hematopoietic stem cell transplantation (HSCT) [3, 9, 10].

Among adult CCS, a substantial percentage—ranging from 21% to 68%—report concerns about fertility potential or perceive a risk of infertility [11, 12, 13, 14, 15, 16]. One study compared CCS to a comparison group without cancer history and found that both female and male CCS worry significantly more about their fertility, but specific risk factors were not analyzed [17]. In other studies of adult CCS, self‐reported fertility‐related distress has been associated with previous gonadotoxic treatment [13], being sexually active or partnered [11, 13, 18], not having children [18], having a desire for (additional) children [18], higher educational attainment [13], and fears about personal health [11]. Women have expressed more concerns than men in some studies [12, 14], but not in others [11, 13]. Additionally, CCS have reported concerns about partner disclosure of possible infertility and worries about the health of future biological children [19, 20, 21].

Despite growing awareness of fertility concerns, population‐based studies of long‐term CCS comparing the prevalence of fertility‐related distress with that of the general population are still lacking. This study addresses this gap by estimating the prevalence and predictors of high fertility‐related distress in a population‐based cohort of young adult CCS aged 19–40 years, with sex‐specific analyses, and by comparing CCS with a sample from the general population.

## 2 Materials and Methods

### 2.1 Participants

This study is part of the Fex‐Can Childhood project, a nationwide population‐based research initiative investigating sexual dysfunction and fertility‐related distress among young adult CCS and a comparison group of similar age [22]. The CCS group included all individuals with a primary cancer diagnosis before the age of 18 and who were 19–40 years old and residing in Sweden at the time of enrollment (1st September 2019). Participants were identified through the National Quality Registry for Childhood Cancer (NQRCC) in Sweden, which records all individuals diagnosed with cancer before age 18. Exclusion criteria included unknown address, difficulty reading or writing Swedish, and self‐reported poor health or cognitive impairments that could hinder participation. Out of 4328 potential participants, 2546 eligible participants completed and returned questionnaires, forming the final study sample (Figure 1). These participants had been diagnosed with cancer between 1981 and 2017. Comparison of responders' and non‐responders' clinical characteristics showed no differences among females [23]. Among males, responders were older at enrollment (mean age 29 and 28 years, respectively; *p* < 0.05) and had a longer time since diagnosis than non‐responders (mean 21 and 20 years, respectively: *p* < 0.05) [23]; however, no differences were observed in cancer diagnoses or treatment intensity [5].

**FIGURE 1:**
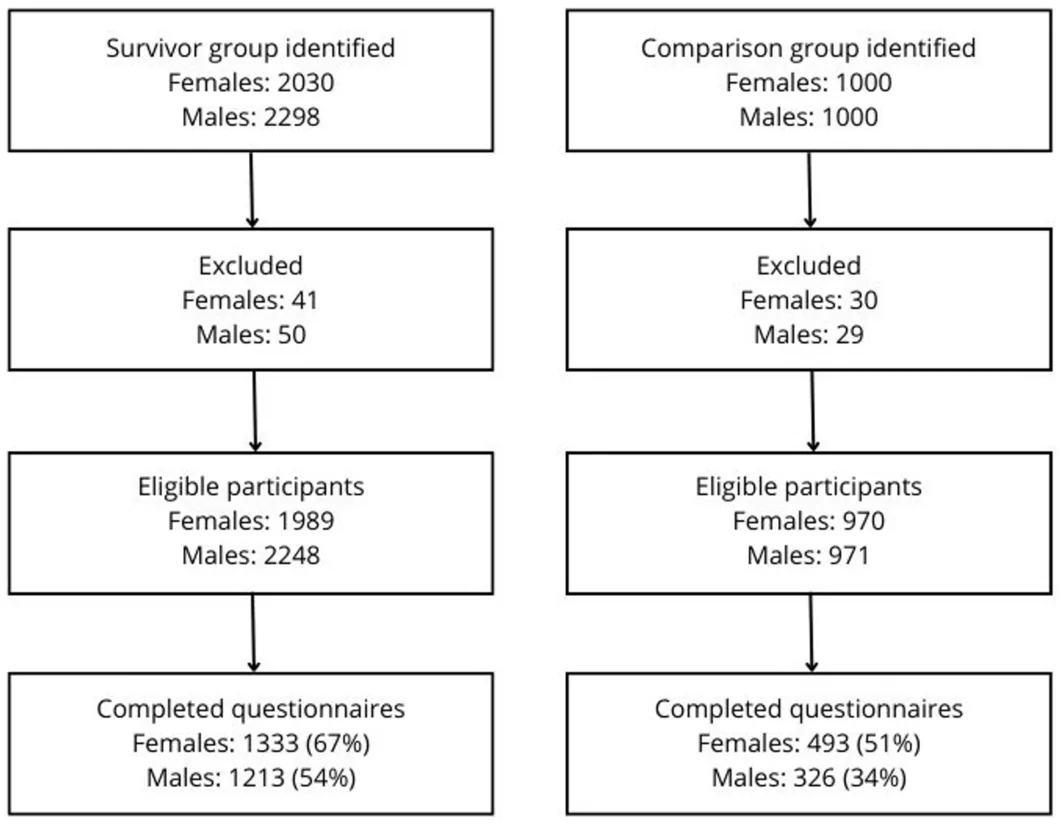
Flow chart showing the number of childhood cancer survivors identified through the National Quality Registry for Childhood Cancer in Sweden and the comparison group identified from the Swedish Population Registry who participated in the study. Exclusion criteria included unknown address, difficulty reading or writing Swedish, self‐reported poor health or cognitive impairments that could hinder participation, and for the comparison group, previous cancer treatment.

A comparison group representing the general population was selected through a random sample of 1000 women and 1000 men, aged 19 to 40 and residing in Sweden at the time of enrollment, identified from the Swedish Population Registry. The same exclusion criteria applied to cancer survivors were used, with the additional exclusion of individuals who reported having received cancer treatment. A total of 30 females and 29 males were excluded. Of the eligible participants, 819 individuals in the comparison group returned the questionnaires (Figure 1). The demographic characteristics of the comparison group were comparable to those of the Swedish general population, except for an underrepresentation of individuals born outside Sweden [24].

### 2.2 Procedure

An information letter describing the study was sent by post to all eligible participants together with a survey and a pre‐paid envelope for survey return. Vital status was verified using a national death registry prior to survey distribution. Participants were given the option to complete the survey via paper, an online platform, or a telephone interview. Two reminders were sent to non‐responders. As an incentive for study participation, two cinema tickets were provided to participants upon completion of the survey. The survey included validated self‐report measures, along with study‐specific questions [22]. Data for CCS were collected between 9/2019 and 2/2020, and for the comparison group 4–6/2018.

Ethical approval was granted by the Regional Ethical Review Board in Stockholm, Sweden (approval numbers: 2015/1609‐31; 2018/2688‐32; 2019/01066; 2019/04603). Written informed consent was obtained from all participants.

### 2.3 Measures

The main outcome measure was the Reproductive Concerns After Cancer (RCAC) scale, a self‐rated measure assessing fertility‐related distress [25]. The RCAC includes six dimensions with three items in each dimension: Fertility potential (concerns about one's ability to have biological children), Partner disclosure (concerns about informing a partner of potential fertility issues), Child's health (concerns about potential health problems in one's biological child, specifically genetic risk), Personal health (concerns about one's future health and ability to raise a child long‐term), Acceptance (difficulty accepting possible infertility), and Becoming pregnant/Achieving pregnancy (concerns about the effort needed to achieve pregnancy). The items are rated on a 5‐point scale (*strongly disagree* to *strongly agree*), and for each dimension, a mean score > 4 is defined as high fertility distress. The comparison group completed only those three RCAC dimensions that are suitable for adults with no history of cancer (Fertility potential, Acceptance, and Becoming pregnant). The RCAC demonstrates satisfactory reliability and construct validity [25, 26, 27]. It has been translated into Swedish for men and women separately [28, 29], with acceptable construct and known‐groups validity confirmed. However, internal consistency was suboptimal for one subscale (Becoming pregnant) [30].

Background data included self‐reported sociodemographics (e.g., birth country, partnership status) and current symptoms of anxiety and depression (Hospital Anxiety and Depression Scale, HADS) [31]. Additionally, CCS were asked to report if they had undergone any fertility preservation (FP) measures.

Clinical data for the survivor group were obtained from the NQRCC and included primary cancer diagnoses, dates of diagnoses, treatment modality and intensity, and relapse status. For each individual, treatment intensity was categorized into four levels (minimally, moderately, very, or most intensive treatments) according to the Intensity of Treatment Rating scale (ITR‐3.0), a psychometrically validated measure based on diagnosis, stage, and treatment data [32].

### 2.4 Statistical Analyses

Group comparisons of the background and clinical variables presented in Table 1 were undertaken using Student's *t*‐test and Chi‐Squared statistics. Differences in the proportion of individuals reporting high fertility‐related distress (mean score > 4 on RCAC dimension) between CCS and the comparison group were assessed with logistic regression, adjusted for sociodemographic characteristics (age at study, education level, being in a relationship, having children, and born in Sweden). To identify predictors of high fertility‐related distress within the survivor group, logistic regression analyses were conducted. The following factors were selected based on previous literature: age at study (19–25, 26–34, 35–40), born in Sweden (yes/no), university degree (yes/no), partner (yes/no), previous children (yes/no), future child wish (yes/no or uncertain), heterosexual (yes/no), HADS anxiety, HADS depression (continuous variables), age at diagnosis (< 9, ≥ 9 years), CNS tumors (yes/no), non‐hematologic, non‐CNS cancers (yes/no), ITR‐3.0 (least or moderate/very or most intensive treatment), undergone FP (yes/no), and ongoing hormone replacement therapy (HRT; yes/no). Only factors significantly associated with high distress in the respective RCAC dimension in bivariate analyses were included in the multivariable logistic regression model. All analyses were undertaken separately for females and males. All tests were two‐tailed and *p* < 0.05 was considered statistically significant.

**TABLE 1:** Sociodemographic, psychological, and clinical characteristics of young adult childhood cancer survivors and a comparison group.

	Survivor group		Comparison group	
	Female (*n* = 1333)	Male (*n* = 1213)	Female (*n* = 493)	Male (*n* = 326)
Sociodemographic characteristics				
Age at study, years, *M* (SD)	28.8 (6.1)*	29.2 (6.1)	29.7 (6.1)	29.3 (6.4)
19–25	455 (34.1)	372 (30.7)	139 (28.4)	111 (34.4)
26–34	583 (43.7)	547 (45.1)	225 (45.9)	126 (39.0)
35–40	295 (22.1)	294 (24.2)	126 (25.7)	86 (26.6)
Country of birth				
Sweden	1272 (95.8)***	1157 (95.9)***	422 (85.6)	271 (83.4)
Highest education				
University	745 (56.1)	523 (43.3)	283 (57.8)	150 (46.3)
Upper secondary	479 (36.1)	589 (48.8)	179 (36.5)	153 (47.2)
Elementary	47 (3.5)	51 (4.2)	18 (3.7)	13 (4.0)
Other	56 (4.2)	45 (3.7)	10 (2.0)	8 (2.5)
In a relationship				
Yes	940 (70.8)***	728 (60.4)***	394 (80.6)	231 (71.5)
Have children				
Yes	495 (37.3)***	368 (30.7)**	230 (46.8)	127 (39.2)
Sexual orientation				
Heterosexual	1178 (89.2)	1137 (94.4)	454 (92.7)	307 (94.8)
Homosexual	20 (1.5)	20 (1.7)	6 (1.2)	7 (2.2)
Bisexual	83 (6.3)	23 (1.9)	26 (5.3)	7 (2.2)
Other^a^	27 (2.0)	11 (0.9)	3 (0.6)	3 (0.9)
Prefer not to answer	13 (1.0)	13 (1.1)	1 (0.2)	—
Psychological outcomes, *M* (SD)				
Anxiety, HADS	8.2 (4.7)**	5.9 (4.1)	7.5 (4.5)	5.5 (3.8)
Depression, HADS	4.4 (3.7)	4.1 (3.4)	4.5 (3.7)	4.0 (3.0)
Clinical characteristics				
Age at diagnosis, years, *M* (SD)	7.4 (5.4)	7.8 (5.4)		
< 9 years of age	779 (58.4)	679 (56.0)		
≥ 9 years of age	554 (41.6)	534 (44.0)		
Diagnostic era				
1981–1989	237 (17.8%)	205 (16.9%)		
1990–1999	585 (43.9%)	550 (45.3%)		
2000–2017	511 (38.3%)	458 (37.8%)		
Time from diagnosis, years, *M* (SD)	20.9 (7.8)	20.9 (7.8)		
Range	1–38	1–37		
Type of cancer^b^				
Hematological cancers	603 (45.2)	615 (50.7)		
CNS tumors	310 (23.3)	267 (22.0)		
Non‐hematologic, non‐CNS cancers	420 (31.5)	331 (27.3)		
Treatment modality				
Chemotherapy	945 (70.9)	887 (73.1)		
Surgery	504 (37.8)	412 (34.0)		
Radiotherapy	282 (21.2)	262 (21.6)		
Cranial irradiation	155 (11.6)	142 (11.7)		
HSCT	83 (6.3)	99 (8.2)		
Intensity of treatment^c^				
Least intensive	156 (11.7)	119 (9.8)		
Moderately intensive	669 (50.2)	613 (50.5)		
Very intensive	330 (24.8)	312 (25.7)		
Most intensive	178 (13.4)	169 (13.9)		
Undergone fertility preservation treatment				
Yes	69 (5.2)^d^	168 (13.9)^e^		
Ongoing hormone replacement therapy				
Yes	121/1295 (9.3)	71/1184 (6.0)		

*Note:* Data indicated as *n* (percentage), unless otherwise indicated. Asterisks denote difference in comparison to females/males in the comparison group.

Abbreviations: CNS, central nervous system; HSCT, hematopoietic stem cell transplantation.

^a^ Includes individuals who reported not knowing, being pansexual, queer, or asexual.

^b^ Diagnostic groups were classified according to the International Classification of Childhood Cancer, third edition (ICCC‐3).

^c^ According to the Intensity of Treatment Rating (ITR‐3.0).

^d^ Cryopreservation of oocytes (*n* = 66) or ovarian tissue (*n* = 3).

^e^ Cryopreservation of sperm (*n* = 166) or testicular tissue (*n* = 2).

* *p* < 0.05.

** *p* < 0.01.

*** *p* < 0.001.

## 3 Results

### 3.1 Participant Characteristics in Survivor and Comparison Group

Sociodemographic, psychological, and clinical characteristics of the study groups are presented in Table 1. Among CCS, the most prevalent diagnoses were hematological cancers, and most had received a moderately intensive treatment regimen. Few CCS had undergone FP; among them, 19% of females and 17% of males had undergone HSCT, while 71% of females and 66% of males had received very or most intensive cancer treatments.

Compared to CCS, a significantly higher proportion of females and males in the comparison group were born outside Sweden, were in a relationship, and had children (Table 1). Female CCS were younger at the time of study and reported higher levels of anxiety than females in the comparison group. There were no significant differences between CCS and their respective comparison group regarding education level, sexual orientation, or depressive symptoms.

### 3.2 Prevalence of High Fertility‐Related Distress, Survivor Versus Comparison Group

Of CCS, 44% of females and 23% of males reported high level of fertility‐related distress in at least one RCAC dimension (Table 2). In females, high distress was most common in the dimensions Fertility potential (23%), Child's health (18%), Acceptance (i.e., acceptance of a life without children; 16%), and Partner disclosure (12%). In male CCS, high distress was relatively equally distributed between Partner disclosure (9%), Fertility potential (8%), Child's health (8%), and Acceptance (8%).

**TABLE 2:** Proportion reporting high fertility‐related distress^a^ in childhood cancer survivors and comparison group by sex; differences between groups were adjusted for sociodemographic characteristics (age at study, education, relationship status, having children, and country of birth).

Dimensions of RCAC	Survivor group		Comparison group		Survivor vs. comparison group^b^	
	Females (*n* = 1333)	Males (*n* = 1213)	Females (*n* = 493)	Males (*n* = 326)	Females	Males
	*n* (%)	*n* (%)	*n* (%)	*n* (%)	Adjusted odds ratio (95% CI)	Adjusted odds ratio (95% CI)
Fertility potential	301 (23)	100 (8)	60 (12)	5 (2)	**1.89 (1.38–2.58)**	**5.13 (2.05–12.84)**
Partner disclosure	156 (12)	113 (9)				
Child's health	240 (18)	101 (8)				
Personal health	58 (4)	26 (2)				
Acceptance	209 (16)	92 (8)	65 (13)	29 (9)	1.05 (0.76–1.44)	0.88 (0.55–1.40)
Becoming pregnant/achieving pregnancy	104 (8)	22 (2)	29 (6)	4 (1)	1.39 (0.89–2.18)	1.42 (0.47–4.26)
High fertility‐related distress in ≥ 1 dimension^a^	584 (44)	282 (23)				
High fertility‐related distress in ≥ 2 dimensions^a^	292 (22)	104 (9)				

*Note:* Statistically significant (*p* < 0.05) differences are indicated in bold.

Abbreviations: CI, confidence interval; RCAC, Reproductive Concerns After Cancer scale.

^a^ A high level of fertility‐related distress is defined as a mean score of > 4 on a dimension.

^b^ The comparison group = reference category.

In logistic regression models adjusting for sociodemographic factors, female CCS were 1.9 times and male CCS 5.1 times more likely to report high distress regarding Fertility potential compared to women and men in the comparison group. No significant group difference was found for the dimensions Acceptance or Achieving pregnancy.

### 3.3 Predictors of High Fertility‐Related Distress in Survivors

In both female and male CCS, the desire to have (additional) children was the strongest predictor of fertility‐related distress, showing a significant association with high distress in four dimensions (Tables 3 and 4). Similarly, not yet having children was significantly associated with high distress, but not as strongly. For females, ongoing HRT was associated with high distress in Fertility potential and Partner disclosure, while in males, it was associated with high distress in most domains. Additionally, having undergone more intensive cancer treatments was significantly associated with high distress in Partner disclosure in both sexes. Having received FP was associated with high distress in Fertility potential in both sexes, but also with Becoming pregnant for females. Furthermore, higher self‐rated anxiety and depression were associated with distress in several RCAC domains for both females and males.

**TABLE 3:** Results of multivariable logistic regression models for high fertility‐related distress in female childhood cancer survivors.

Predictors	Dimensions of RCAC											
	Fertility potential		Partner disclosure		Child's health		Personal health		Acceptance		Becoming pregnant	
	OR	95% CI	OR	95% CI	OR	95% CI	OR	95% CI	OR	95% CI	OR	95% CI
Age at study (in years)												
26–34 vs. 19–25 (ref)	0.98	0.70–1.37	**0.50**	**0.33–0.78**					0.77	0.54–1.11	1.18	0.74–1.88
35–40 vs. 19–25 (ref)	1.02	0.58–1.76	0.68	0.34–1.35					0.58	0.28–1.20	0.84	0.39–1.81
Country of birth												
Sweden vs. other (ref)	**2.54**	**1.03–6.30**							**3.77**	**1.12–12.63**		
Relationship status												
Partner vs. no partner (ref)			**0.56**	**0.38–0.83**								
Having children												
No vs. yes (ref)	**1.48**	**1.00–2.17**	**3.32**	**1.71–6.42**	**0.54**	**0.40–0.72**	**0.23**	**0.13–0.43**	**2.80**	**1.72–4.57**	1.28	0.76–2.15
Wish for (more) children												
Yes vs. no (ref)	**8.86**	**5.75–13.64**	**2.40**	**1.51–3.81**			0.62	0.34–1.12	**18.71**	**8.96–39.06**	**2.40**	**1.44–4.00**
Sexual orientation												
Heterosexual vs. other (ref)												
Anxiety (HADS)	**1.11**	**1.06–1.15**	**1.11**	**1.06–1.17**	**1.13**	**1.09–1.18**	**1.24**	**1.15–1.34**	1.03	0.98–1.07	1.05	0.99–1.11
Depression (HADS)	0.99	0.94–1.04	1.04	0.98–1.10	0.99	0.95–1.05	1.03	0.95–1.12	**1.06**	**1.00–1.12**	**1.09**	**1.02–1.16**
Type of cancer												
CNS tumors vs. hematological cancer (ref)	**0.62**	**0.42–0.94**										
Non‐hematological, non‐CNS vs. Hematological cancer (ref)	0.95	0.68–1.34										
Treatment intensity												
Very/most intensive vs. least/moderately intensive (ref)	1.32	0.96–1.83	**1.52**	**1.02–2.26**								
Fertility preservation												
Yes vs. no (ref)	**3.28**	**1.81–5.93**	1.41	0.72–2.78	1.71	0.96–3.08			1.75	0.95–3.21	**2.42**	**1.22–4.79**
Ongoing hormone replacement therapy												
Yes vs. no (ref)	**2.01**	**1.27–3.45**	**3.44**	**2.01–5.90**								

*Note:* Statistically significant predictors in the multivariable model (*p* < 0.05) are indicated in bold. Each model included the predictors that were significantly associated with high distress in the respective RCAC dimension in bivariate analyses. Education (university degree vs. other) and age at diagnosis (< 9 vs. ≥ 9 years) were not significantly associated with high distress in any RCAC dimension and were therefore not included in the regression models.

Abbreviations: CI, Confidence Interval; CNS, Central nervous system; OR, Odds Ratio; RCAC, Reproductive Concerns After Cancer scale; ref, reference category.

**TABLE 4:** Results of multivariable logistic regression models for high fertility‐related distress in male childhood cancer survivors.

Predictors	Dimensions of RCAC											
	Fertility potential		Partner disclosure		Child's health		Personal health		Acceptance		Achieving pregnancy	
	OR	95% CI	OR	95% CI	OR	95% CI	OR	95% CI	OR	95% CI	OR	95% CI
Age at study (in years)												
26–34 vs. 19–25 (ref)	0.91	0.55–1.50	0.86	0.53–1.41					0.81	0.49–1.33		
35–40 vs. 19–25 (ref)	1.25	0.61–2.56	1.10	0.55–2.21					0.77	0.37–1.64		
Country of birth												
Sweden vs. other (ref)					**0.35**	**0.16–0.78**						
Education												
No university degree vs. university degree (ref)							**3.43**	**1.19–10.64**				
Relationship status												
Partner vs. no partner (ref)			**0.40**	**0.25–0.66**								
Having children												
No vs. yes (ref)	**3.00**	**1.39–6.46**	**4.42**	**1.63–11.97**					1.77	0.89–3.52		
Wish for (more) children												
Yes vs. no (ref)	**6.13**	**3.25–11.57**	**3.32**	**1.97–5.60**					**5.58**	**2.94–10.66**	**3.32**	**1.20–9.24**
Anxiety (HADS)	**1.10**	**1.03–1.17**	**1.11**	**1.04–1.18**	**1.14**	**1.08–1.21**	**1.17**	**1.06–1.30**	1.04	0.97–1.10	1.11	0.99–1.25
Depression (HADS)	1.07	1.00–1.15	**1.10**	**1.03–1.18**	1.05	0.98–1.12	1.06	0.94–1.19	**1.08**	**1.01–1.17**	1.05	0.92–1.20
Age at diagnosis (in years)												
< 9 vs. ≥ 9 (ref)					**0.64**	**0.42–0.99**						
Treatment intensity												
Very/most intensive vs. least/moderately intensive (ref)	1.28	0.80–2.07	**1.66**	**1.06–2.62**	**0.62**	**0.39–0.98**						
Fertility preservation												
Yes vs. no (ref)	**1.89**	**1.08–3.30**										
Ongoing hormone replacement therapy												
Yes vs. no (ref)	**3.82**	**1.89–7.71**	**4.67**	**2.44–8.95**			**3.43**	**1.19–9.89**			**3.79**	**1.21–11.92**

*Note:* Statistically significant predictors in the multivariable model (*p* < 0.05) are indicated in bold. Each model included the predictors that were significantly associated with high distress in the respective RCAC dimension in bivariate analyses. Sexual orientation (heterosexual vs. other) and type of cancer (CNS tumors vs. hematological cancer; non‐hematological, non‐CNS vs. hematological cancer) were not significantly associated with high distress in any RCAC dimension and were therefore not included in the regression models.

Abbreviations: CI, Confidence Interval; CNS, Central nervous system; OR, Odds Ratio; RCAC, Reproductive Concerns After Cancer scale; ref, reference category.

## 4 Discussion

In this large population‐based cohort, fertility‐related distress was common among young adult survivors of childhood cancer, particularly among females. Both female and male survivors most frequently worried about their ability to have children and were significantly more likely to report high distress about their fertility potential than their general population peers. A desire for (additional) children was the factor most strongly associated with high fertility‐related distress, especially in female survivors, followed by ongoing HRT and not yet having children. In addition, more intensive treatment regimens, having received FP, as well as higher self‐reported anxiety and depression were associated with high fertility‐related distress.

These findings align with previous research showing that reproductive concerns are prevalent among CCS [19, 33]. The proportion of high fertility‐related distress was relatively similar to that reported in a slightly older cohort of young adults diagnosed with cancer in adulthood (≥ 18 years) and assessed 1.5 years post‐diagnosis [34]. However, survivors of young adult cancer [34] reported greater distress regarding both their own and their child's health compared to CCS. This may reflect the shorter time since diagnosis and the higher proportion of survivors of young adult cancer already having children, which could shift their focus from concerns about fertility to worries about their child's well‐being and their own health.

In general, female survivors report more long‐term psychosocial sequelae than men [21]. Although previous studies on sex differences in fertility‐related distress have shown mixed results [12, 13, 14, 34], in the present study women reported such distress to a higher extent than men, among both the survivors and the general population sample, indicating that women are overall more aware and concerned of fertility issues. Nevertheless, male CCS demonstrated a significantly greater impact, exhibiting five times the odds of experiencing high distress about their fertility potential compared to their male counterparts in the comparison group. One possible explanation is that, among the particularly affected subgroup of males requiring testosterone replacement, fertility may have been addressed during follow‐up visits and at the initiation of HRT due to testosterone deficiency, thereby heightening fertility‐related concerns across several RCAC dimensions and rendering fertility a more pertinent concern for those male survivors compared to men in the general population. Further, this finding highlights the importance of sex‐stratified analyses using corresponding comparison groups.

Despite the well‐documented risk of infertility following high‐intensity cancer treatment [8], reproductive preferences, sociodemographic characteristics, and concurrent psychological factors showed stronger associations with self‐reported fertility‐related distress than overall treatment intensity. This finding may partly reflect the use of the ITR‐3.0, a psychometrically tested and validated approach to classifying overall pediatric cancer treatment intensity that does not specifically capture gonadotoxic potential. However, the associations found in this study may also reflect that treatment‐related infertility risk, although well documented, may not always be effectively communicated to or fully understood by patients and their families.

The desire for (additional) children was the factor most strongly associated with fertility‐related distress, particularly in the Fertility potential and Acceptance dimensions, consistent with previous research [18]. High scores in these dimensions may partly reflect the importance that individuals place on future biological parenthood rather than ineffective coping or poor distress management. Conversely, low distress may reflect a lower priority placed on biological parenthood and should not necessarily be interpreted as better psychological adjustment. Although CCS have a lower probability of partnering in adulthood [35], which was also seen in the present study, having no current partner was significantly associated only with higher distress concerning disclosure of one's infertility to a partner.

Having undergone FP was associated with high distress regarding fertility potential in both women and men, and with high distress about achieving pregnancy among females. Rather than indicating that FP increases distress, receipt of FP may identify survivors who were informed about their potential infertility risk. Differences in FP practices may also be relevant: In Sweden sperm cryopreservation has been routinely offered within the public healthcare system to post‐pubertal males for several decades and was standard practice at the time of cancer diagnosis even in early treatment eras, explaining its predominance in the present data. In contrast, invasive oocyte harvesting in post‐pubertal females has become available only in recent decades. Consequently, FP among females in the present cohort has predominantly been offered later in life and mainly to survivors who received more intensive cancer treatments. This may increase awareness of potential infertility and contribute to increased distress among female survivors. In survivors of young adult cancer in Sweden, FP has similarly been linked to high distress concerning fertility potential, but this was observed only among female survivors [34]. These findings highlight the need for support and counseling to help CCS manage fertility‐related concerns as they reach reproductive age. This need is further underscored by the association between higher self‐rated anxiety and depression with fertility‐related distress, emphasizing the importance of comprehensive mental health support.

### 4.1 Limitations

This population‐based cohort of CCS was identified from a national quality registry. Nevertheless, the low response rates among controls and male survivors may have introduced selection bias, limiting the generalizability of the findings, particularly for men. Male CCS participants were older and had a longer follow‐up time since diagnosis than non‐participants. No other indications of response bias related to the available clinical variables were identified. Comparisons between survivors and controls were limited to the three RCAC dimensions applicable to individuals without a history of cancer.

This sample comprised patients treated for cancer 1981–2017. Both cancer treatments and FP have advanced in the past decades, which should be taken into consideration. Moreover, the lack of detailed treatment‐exposure data prevented classification of gonadotoxic risk according to established treatment‐specific criteria. Finally, this study did not assess if fertility‐related distress aligned with the survivors' gonadal functioning or their knowledge about their fertility status.

### 4.2 Conclusions

The present findings underscore the importance of addressing distress related to fertility potential within childhood cancer survivorship care, taking into account survivors' reproductive preferences, previous and ongoing treatments, and concurrent psychological symptoms. Survivors experiencing fertility‐related distress may benefit from support addressing both general symptoms of anxiety and depression as well as the unique fertility‐related concerns that CCS face. Such support could be provided through multidisciplinary collaboration, involving oncologists, specialized nurses, fertility specialists, and mental health professionals within long‐term follow‐up care.

## Author Contributions

**Anu Haavisto:** writing – original draft, writing – review and editing. **Kirsi Jahnukainen:** writing – review and editing, supervision, funding acquisition. **Poorna Anandavadivelan:** formal analysis, writing – review and editing. **Emma Hovén:** formal analysis, writing – review and editing. **Lena Wettergren:** conceptualization, methodology, resources, writing – review and editing, supervision, project administration, funding acquisition. **Claudia Lampic:** conceptualization, methodology, resources, writing – review and editing, project administration, supervision, funding acquisition.

## Funding

This work was supported by The Swedish Cancer Society (CAN 2013/886, CAN 2016/615, 190196Pj, 222311Pj), the Swedish Childhood Cancer Foundation (TJ2014‐0050, TJ2019‐0045, PR2014‐0177, PR2016‐0075, PR2017‐ 0037 and KP2020‐0012), the Swedish Research Council for Health, Working Life and Welfare (2014‐4689, 2019‐00839), the Swedish Research Council (2017‐01530, 2022‐00832), the Cancer Research Foundations of Radiumhemmet (161272, 221363), the Birgitta and Carl‐Axel Rydbeck's Research Grant for Paediatric Research (2020‐00335 and 2021‐00079), The Cancer Foundation Finland, and the Finnish Foundation of Paediatric Research.

## Conflicts of Interest

The authors declare no conflicts of interest.

## Data Availability

The data that support the findings of this study are available from the corresponding author upon reasonable request.
